# Decompressive Hemicraniectomy Associated With Ultrasound-Guided Minimally Invasive Puncture and Drainage Has Better Feasibility Than the Traditional Hematoma Evacuation for Deteriorating Spontaneous Intracranial Hemorrhage in the Basal Ganglia Region: A Retrospective Observational Cohort Study

**DOI:** 10.3389/fneur.2020.561781

**Published:** 2021-01-12

**Authors:** Yuan Cheng, Jin Chen, Guanjian Zhao, Zongyi Xie, Ning Huang, Qiang Yang, Weifu Chen, Qin Huang

**Affiliations:** Department of Neurosurgery, The Second Affiliated Hospital of Chongqing Medical University, Chongqing, China

**Keywords:** spontaneous intracerebral hemorrhage, intracranial pressure, decompressive hemicraniectomy, minimally invasive puncture and drainage, peri-hematoma edema

## Abstract

**Objectives:** Spontaneous intracerebral hemorrhage (ICH) is a devastating disease with higher mortality and disability rates; however, ideal surgical management is still to be determined for critical ICH. The purpose of this study was to prove the feasibility and unique clinical value of a novel combination, decompressive hemicraniectomy associated with ultrasound-guided minimally invasive puncture and drainage (DH + MIPD), for deteriorating ICH in the basal ganglia region.

**Methods:** According to the enrollment criteria, 168 ICH patients were analyzed retrospectively, of which 86 patients received DH + MIPD and 82 patients received DH associated with traditional hematoma evacuation as the control group. The change process of three parameters, including hematoma size, peri-hematoma edema, and intracranial pressure (ICP), in a period of time after operation, as well as the short- and long-term therapeutic effect, was compared.

**Results:** The DH + MIPD method could effectively achieve the evacuation rate of hematoma up to 87% at 5 days post-operation and had the significant advantages of minimal injury to cerebral tissue, less degree of edema, better effect of decreasing ICP, shorter operation time, less blood loss, and lower mortality compared with the control method. The DH + MIPD group had a significantly higher survival rate within 1 year post-operation (*P* = 0.007) and better functional outcome at 90 and 180 days post-operation (*P* = 0.004). A subgroup analysis pointed out that the DH + MIPD method had a definite survival advantage for critical ICH patients older than 60 years old and with hematoma located in the left dominant hemisphere.

**Conclusions:** Our results proved the better feasibility of DH + MIPD on hematoma evacuation and implicated its significant advantages of reducing mortality and improving functional recovery. This method provides one more choice for the individualized therapy of ICH in the basal ganglia region.

## Introduction

Spontaneous intracerebral hemorrhage (ICH) is a common and devastating disease, with an incidence of 24.6 per 100,000 person-years and up to 40.4% mortality in the first 30 days; especially, more than half of the survivors with different degrees of neurological deficit have seriously aggravated the burden on families and society (1). Although great progress in the diagnosis and treatment of ICH has been made over the recent decades, there is still no definite and ideal therapeutic method at present.

Intuitively, hematoma evacuation may have many theoretical advantages of alleviating intracranial pressure (ICP), easing mass effect, preventing cerebral herniation, and mitigating the neurotoxic effects of clot components, including thrombin, hemoglobin degradation products, and inflammatory cascades (2). However, during the process of hematoma evacuation, medical appliances must go through the normal cerebral tissue to reach the deeper hematoma, such as in the basal ganglia region, which would inevitably cause iatrogenic injury to a certain extent. Meanwhile, the operations are always objectively accompanied with risks and complications. At present, the general methods of hematoma evacuation include craniotomy, decompressive hemicraniectomy (DH) + hematoma evacuation, neuro-endoscopy, and minimally invasive puncture and drainage (MIPD). Despite a great deal of comparison and research on these surgical methods, ideal surgical management is still to be determined. Two large, multicenter, multinational, randomized clinical trials, the International Surgical Trial in Intracerebral Hemorrhage (STICH) and the STICH II, have shown that early surgical evacuation could not improve the survival rate or functional outcome when compared to the best medical management for ICH patients (3, 4). Perhaps this result would discourage all the neurosurgeons, but in these two trials, patients who were considered to definitely benefit from surgical evacuation were not in the inclusion criteria of the study (5).

Therefore, for those serious ICH patients with deteriorating consciousness or at an early stage of cerebral herniation, there is no doubt that an operation is the only life-saving method. How to choose the operative strategy? Is there an ideal method that can most effectively reduce ICP while minimizing iatrogenic cerebral injury during hematoma evacuation and finally ameliorating the functional prognosis of patients? Based on this thought, we made a research on a new combination, DH associated with ultrasound-guided MIPD (DH + MIPD), followed by thrombolytic therapy with urokinase, then investigated the change process of hematoma, peri-hematoma edema (PHE), and ICP in a period of time post-operation, and finally evaluated the short- and long-term therapeutic effect of this new form. Through analysis of these data, we intended to demonstrate the feasibility and unique clinical value of the DH + MIPD method and provide one more choice for the individualized treatment of ICH in the basal ganglia region.

## Methods

### Patients

The data of patients with ICH in the basal ganglia region from January 1, 2016 to December 31, 2018 were analyzed in this retrospective cohort trial. This study was approved by the Ethics Committee of the Second Affiliated Hospital of Chongqing Medical University. Written informed consent was obtained from all patients whose clinical files were used in the present study. All ICH patients were admitted to the hospital within 24 h of the ictus. According to the diagnostic criteria of the guidelines for the primary prevention of stroke (6), all diagnoses of ICH were confirmed by computed tomography (CT). Subsequently, CT angiogram was arranged to exclude potential cerebrovascular pathology.

The inclusion criteria were as follows: (1) all patients were operated on within 24 h after the ictus, (2) hematoma volume (HV) ≥40 cm^3^ and midline shift ≥5 mm, (3) hematoma located in the basal ganglia, with (or without) the brain ventricle, (4) pre-operative Glasgow Coma Scale (GCS) score ≤8 or GCS >8 with obviously progressive deterioration of consciousness, (5) age: 18–80 years, and (6) pupils: one enlarging at the same side of the hematoma or both of normal size with obviously progressive deterioration of consciousness during admission.

The exclusion criteria were as follows: (1) ICH induced by intracranial aneurysms, arteriovenous malformations, tumors, infarction, or trauma, (2) hematoma located in other places, such as the lobes, brainstem, and cerebellum, (3) a prior history of stroke with neurological deficits, (4) complicated with severe cardiac, hepatic, renal, or pulmonary disease or functional failure, (5) antithrombotic drugs usage or coagulopathy, including thrombocytopenia and hepatitis, and (6) both pupils are dilated.

Finally, there were 168 patients analyzed, of which 86 cases (admitted in ward I) were treated with the DH + MIPD method and 82 cases (admitted in ward II) received DH associated with traditional hematoma evacuation as the control group. The CT scans were arranged routinely at 1, 3, 5, 7, 14, and 21 days post-operation and within 24 h after the removal of the ICH catheter. If any patient's condition deteriorated suddenly, emergency CT would be arranged immediately.

### Interventions

#### DH + MIPD Group

**Figure 1:**
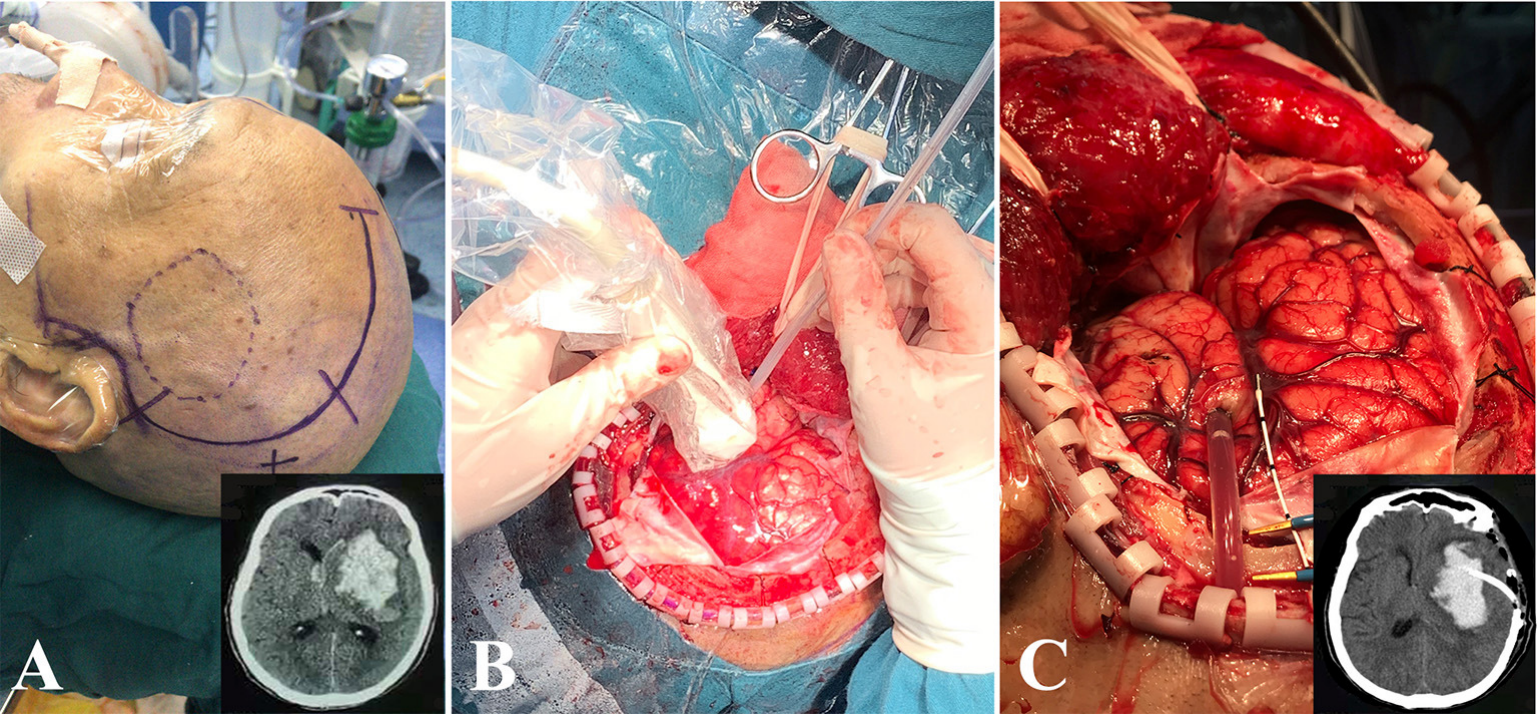
The process of DH + MIPD method. **(A)** The scope of scalp incision of DH + MIPD and CT pre-operation (lower-right corner). **(B)** Under the guidance of ultrasound, a drainage catheter was implanted into the cavity of the hematoma. **(C)** After satisfactory hematoma aspiration, the drainage catheter was fixed properly, and then the ICP probe would be placed into the hematoma cavity, 2–3 cm below the cortex. The CT post-operation is shown in the lower-right corner. DH + MIPD, decompressive hemicraniectomy + minimally invasive puncture and drainage; ICP, intracranial pressure.

All operations were performed under general anesthesia. The procedure was similar to the method described by Zhou et al. (8). DH was performed in the fronto-temporo-parietal region of each case, and a large bone flap with a diameter of at least 10 ^*^ 10 cm^2^ was removed. After cutting and suspending the dura, ultrasound (LOGIQ E9, GE, Boston, MA, USA) was used to detect the size of hematoma in real time. Under the guidance of ultrasound, a drainage catheter with a diameter of 2.6 mm (no. 91503, Medtronic, Minneapolis, USA) was implanted and reached a depth of 1/2–2/3 of the diameter of the hematoma cavity. Then, a hematoma aspiration was carefully carried out freehand using a 10-ml syringe. Generally, 1/3–1/2 of HV was aspirated, and once resistance was felt during this process, it should be stopped immediately. Subsequently, the catheter was tunneled subcutaneously away from the incision and then connected to a three-way stopcock and a closed drainage system. Finally, an ICP probe (Codman, Johnson & Johnson, New Brunswick, USA) was placed into the hematoma cavity, 2–3 cm below the cortex. The whole process of this operation is shown in Figure 1.

At 6 h later, after confirming the positioning of the catheter and no new bleeding in the hematoma cavity with post-operative CT, urokinase (2.0 × 10^4^ IU; Livzon, Zhuhai, Guangdong, China) was administrated into the clot every 12 h for up to nine doses or until the residual HV was ≤10 cm^3^. After each dose, we clamped the catheter for 2 h and then re-opened it to gravity drainage for 10 h. The retention of the catheter and the ICP probe usually did not exceed 6 days.

#### Control Group

The manipulation of the bone flap and the dura mater was the same as that of the DH + MIPD group. Thereafter, a trans-cortical approach to the clot was performed from the sulcus between the superior and the middle temporal gyrus or through lateral fissure, and the clot was evacuated with the assistance of an operative microscope (M5250H4, Leica, Wetzlar, Germany) through standard neurosurgical techniques. Finally, the ICP probe was placed the same way as that in the DH + MIPD group.

### Evaluation of Therapeutic Effect

The therapeutic effect was evaluated in two parts, the short-term outcomes and the long-term outcomes. The short-term outcomes included the following: (1) HV and evacuation rate (ER) of the hematoma at 1, 3, and 5 days post-operation, ER = (pre-operative HV – residue HV) / pre-operative HV ^*^ 100%, (2) edema volume (EV) and the relative increasing rate (RIR) of edema at 1, 3, 5, 7, 14, and 21 days post-operation, RIR of edema = (current EV - pre-operative EV)/pre-operative EV ^*^ 100%, (3) ICP within the first 5 days post-operation, (4) some clinical parameters related to surgery: operative time, blood loss in operation, treatment duration in ICU, and common complications (such as re-bleeding, epilepsy, intracranial infection, pulmonary infection, renal failure, upper gastrointestinal bleeding, and mortality within 30 days post-operation). With the help of the 3D Slicer software (7), the 3D models of the hematoma and PHE could be constructed. Meanwhile, the corresponding volume values could also be accurately provided.

The long-term outcomes included the following: (1) comparison of survival percentage and survival advantage between the two groups and (2) comparison of functional outcome: modified Rankin Scale (mRS) assessments within 90 and 180 days post-operation, with mRS 0–3 as favorable outcomes *vs*. mRS 4–6 as unfavorable outcomes.

### Statistical Analysis

All statistical analyses were done using SPSS 23.0 software package (SPSS Inc., Chicago, IL, USA). Values are expressed as mean ± SD. The quantitative data were analyzed by two-sample *t*-test, while the categorical data were compared using chi-squared test. The repeated-measurement data were analyzed by two-way ANOVA, while the ordered-grade data were compared using Mann–Whitney *U*-test. A *P* < 0.05 was considered as statistically significant.

### Data Availability

All relevant data are presented within the article and its supporting information files. Additional information can be obtained upon request to the corresponding author.

## Results

The baseline characteristics of the 168 ICH patients are shown in Table 1. There were no significant differences in each parameter between the two groups (*P* > 0.05).

**Table 1:** Baseline characteristics of the 168 intracerebral hemorrhage patients.

	**DH + MIPD group (*n* = 86)**	**Control group (*n* = 82)**	***P***
Age	57.9 ± 10.5	59.3 ± 11.6	0.31
≤60 years	39 (45.3)	34 (41.5)	0.61
>60 years	47 (54.7)	48 (58.5)	
Gender: female/male	46/40 (53.5/46.5)	39/43 (47.6/52.4)	0.44
Location: left/right	50/36 (58.1/41.9)	48/34 (58.5/41.5)	0.96
SBP (mmHg)	178.9 ± 20.6	180.3 ± 17.1	0.63
GCS	7.2 ± 2.0	7.5± 2.0	0.73
9–12	18 (20.9)	22 (26.8)	0.37
3–8	68 (79.1)	60 (73.2)	
HV (cm^3^)	65.6 ± 14.0	64.8 ± 13.3	0.32
≤ 60 cm^3^	41 (47.7)	43 (52.4)	0.54
>60 cm^3^	45 (52.3)	39 (47.6)	
Time from ictus to operation (h)	7.7 ± 3.8	7.9 ± 3.5	0.36

*Values are presented as mean ± SD or number (%)*.

*DH + MIPD, decompressive hemicraniectomy + minimally invasive puncture and drainage; SBP, systolic blood pressure; GCS, Glasgow Coma Scale; HV, hematoma volume*.

### Comparisons of Short-Term Therapeutic Effects

#### Change Process of Hematoma in Both Groups

**Figure 2:**
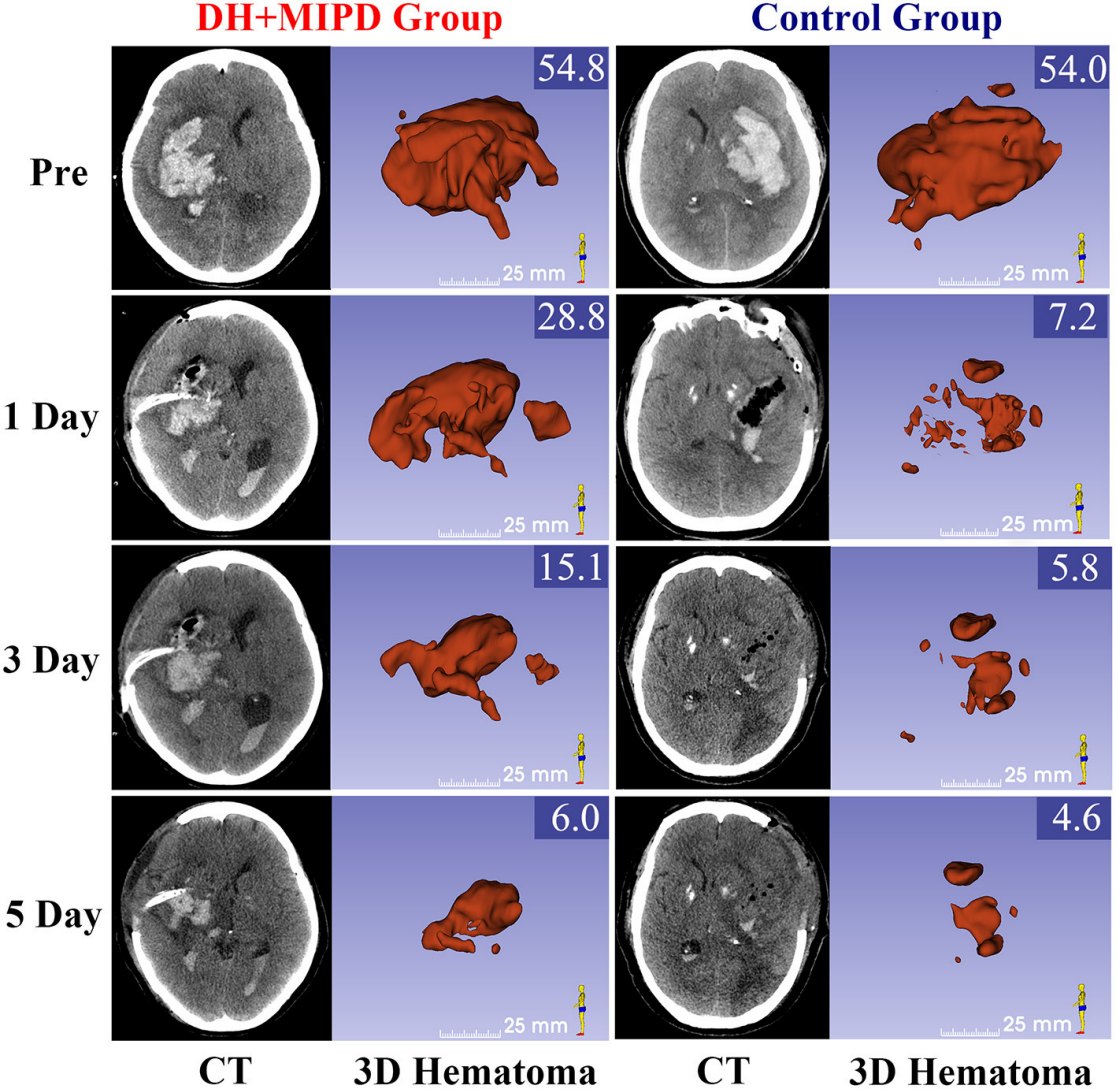
CT scans and the corresponding 3-D hematoma of the DH + MIPD group and the control group on pre-operation and at 1, 3, and 5 days post-operation are shown. The values of HV are marked in the upper-right corner of the 3-D images (cm^3^). DH + MIPD, decompressive hemicraniectomy + minimally invasive puncture and drainage; HV, hematoma volume.

The CT scans combined with the corresponding 3-D hematoma were more apt to directly present the characteristics of hematoma evacuation between the two surgical methods (Figure 2). According to residual HV and ER at different time points (**Figures 4A,B**), the change process of hematoma was obviously different in both groups (*P* = 0.000). For the control group, nearly 83% of hematoma could be directly removed during an operation. By contrast, the characteristic of hematoma evacuation was a gradual process in the DH + MIPD group. Under the action of urokinase, the ER of hematoma in the DH + MIPD group could effectively reach up to 87% at 5 days post-operation from 44% at 1 day post-operation.

#### Change Process of PHE in Both Groups

**Figure 3:**
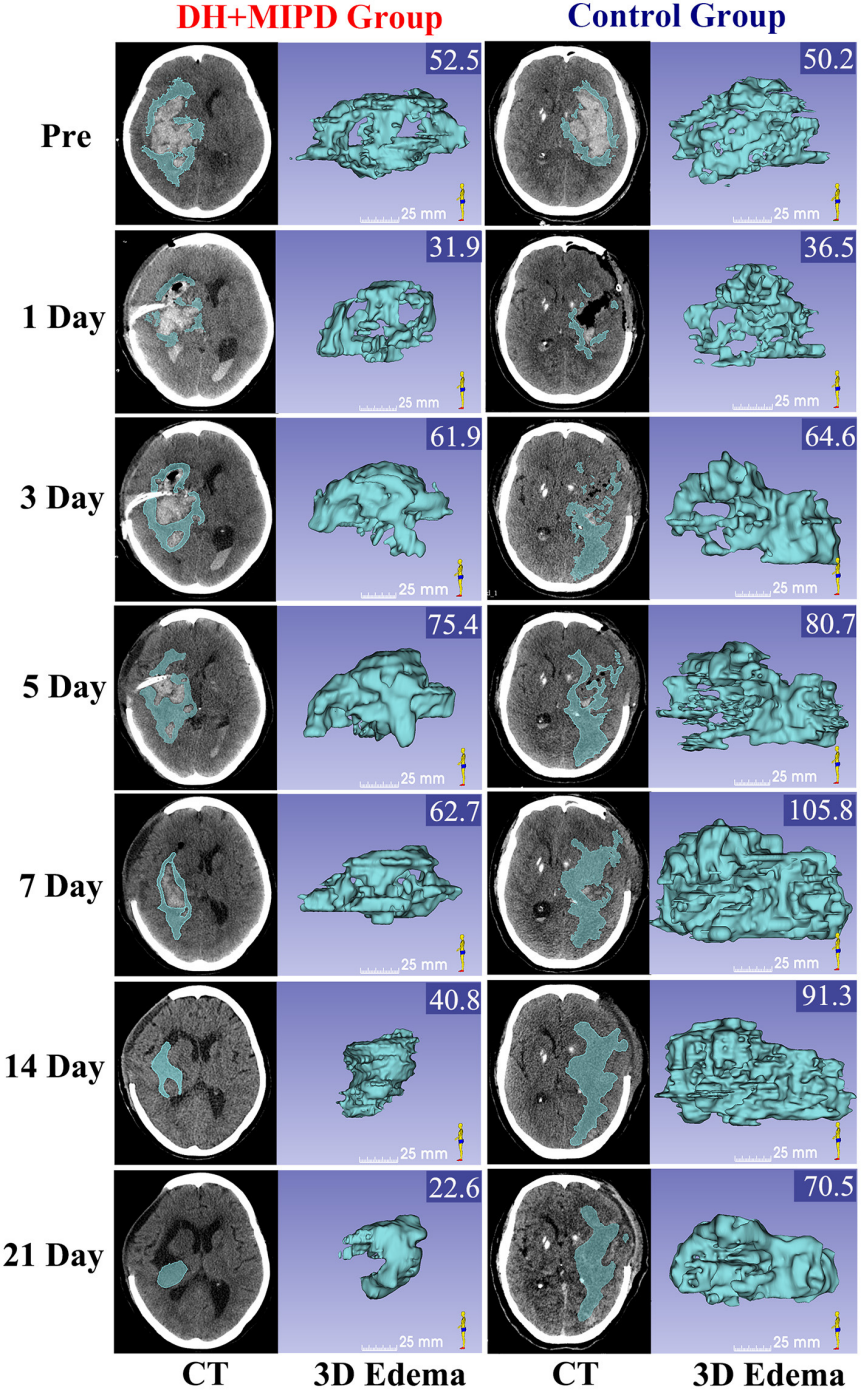
CT scans and the corresponding 3-D edema of the DH + MIPD group and the control group on pre-operation and at 1, 3, 5, 7, 14, and 21 days post-operation are shown. The values of EV were marked in the upper right corner of the 3-D images (cm^3^). DH + MIPD, decompressive hemicraniectomy + minimally invasive puncture and drainage; EV, edema volume.

**Figure 4:**
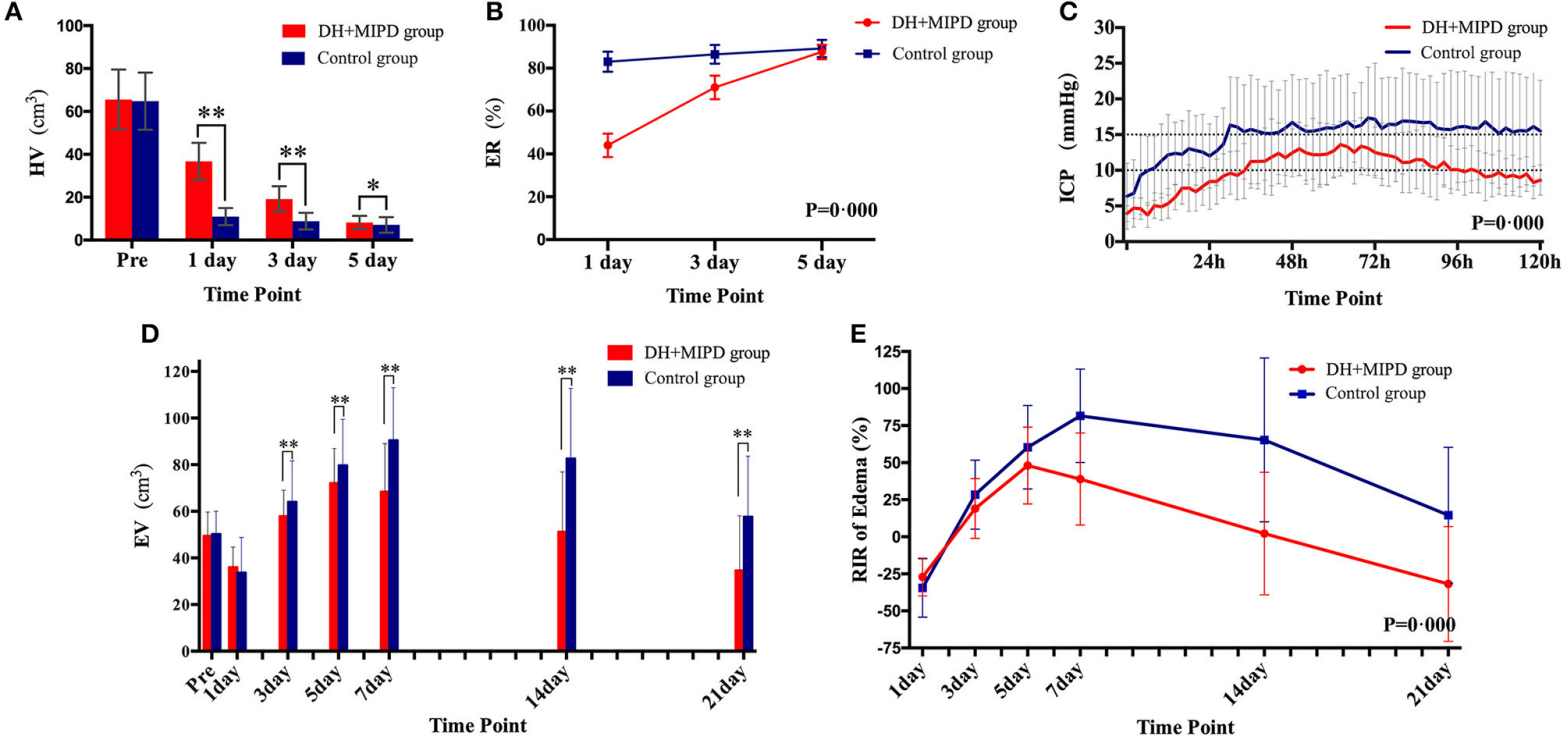
Comparison of HV **(A)**, ER **(B)**, ICP **(C)**, EV **(D)**, and RIR of edema **(E)** between the DH + MIPD group and the control group within a certain period of time post-operation. DH + MIPD, decompressive hemicraniectomy + minimally invasive puncture and drainage; HV, hematoma volume; ER, evacuation rate, ER = (pre-operative HV – residue HV) / pre-operative HV *100%; ICP, intracranial pressure; EV, edema volume; RIR, relative increasing rate; RIR of edema = (current EV - pre-operative EV)/pre-operative EV * 100%. The symbols * and ** indicate *P* < 0.05 and *P* < 0.01, respectively. Each bar represents the mean ± SD.

The 3-D PHE structures of the two groups at different time points were heterogeneous and irregular (Figure 3). According to the EV and the RIR of PHE at different time points (Figures 4D,E), the change process of PHE was also obviously different in both groups (*P* = 0.000). After a short lesson post-operation, the RIR of PHE in both groups increased fastest in the first 3 days. Afterwards, the PHE in the DH + MIPD group continued to grow at a slower rate, reaching its peak of 72.156 ± 1.877 cm^3^ at 5 days post-operation, and then quickly decreased to 34.693 ± 2.661 cm^3^ at 21 days post-operation. In comparison, the PHE in the control group kept going up to 90.473 ± 2.385 cm^3^ at 7 days post-operation, then went down slowly but was maintained at a higher level for about 1 week, and finally decreased obviously to 57.674 ± 2.725 cm^3^ at 21 days post-operation.

#### Change Process of ICP in Both Groups

The changes of ICP in both groups were significantly different within the first 5 days post-operation, and the mean ICP of the DH + MIPD group, 9.89 ± 0.46 mmHg, was lower than that of the control group, 14.84 ± 0.47 mmHg (*P* = 0.000) (Figure 4C). The ICP of the DH + MIPD group increased gradually, exceeded 10 mmHg at 2 days post-operation, reached a peak of 13.6 mmHg at 3 days post-operation, and then slowly declined to finally below 10 mmHg at 5 days post-operation. By contrast, the ICP of the control group ascended quickly and exceeded 15 mmHg at 2 days post-operation and then kept on fluctuating above this level (maximum, 17.3 mmHg) for the following 3 days.

#### Comparison of the Clinical Parameters Related to Operation Between the Two Groups

The results related to operation between the two groups are shown in Table 2. The values of the mean operative time, mean blood loss, and length in ICU in the DH + MIPD group were significantly lower than those in the control group, respectively (177.0 ± 20.6 vs. 257.7 ± 31.7 min, 183.4 ± 26.1 vs. 344.5 ± 73.0 ml, 3.6 ± 0.4 vs. 5.9 ± 0.4 days, *P* < 0.001). The mortality within 30 days post-operation in both groups was significantly different, with 14% in the DH + MIPD group and 26.8% in the control group (*P* = 0.038). There were no significant differences about operation-related complications between the two groups.

**Table 2:** Comparison of the operation-related parameters and the functional outcomes of the two groups.

**Clinical parameter**	**DH + MIPD group (*n* = 86)**	**Control group (*n* = 82)**	***P***
Operative time (min)	177.0 ± 20.6	257.7 ± 31.7	<0.001
Blood loss during operation (ml)	183.4 ± 26.1	344.5 ± 73.0	<0.001
Treatment duration in ICU (day)	3.6 ± 0.4	5.9 ± 0.4	<0.001
**COMPLICATIONS**			
Re-bleeding	2 (2.3%)	5 (6.1%)	0.403
Epilepsy	3 (3.5%)	7 (8.5%)	0.291
Intracranial infection	2 (2.3%)	6 (7.3%)	0.248
Pulmonary infection	26 (30.2%)	31 (37.8%)	0.300
Renal failure	6 (8.1%)	8 (9.8%)	0.515
Upper gastrointestinal bleeding	8 (9.3%)	11 (13.4%)	0.431
30 days mortality	12 (14.0%)	22 (26.8%)	0.038
90 days mortality	17 (19.8%)	28 (34.1%)	0.035
180 days mortality	19 (22.1%)	31 (37.8%)	0.026
90 days mRS			0.004
Favorable	39 (45.3)	20 (24.4)	
Unfavorable	47 (54.7)	62 (75.6)	
180 days mRS			0.004
Favorable	43 (50.0)	23 (28.0)	
Unfavorable	43 (50.0)	59 (72.0)	

*Values are presented as mean ± SD or number (%)*.

*DH + MIPD, decompressive hemicraniectomy + minimally invasive puncture and drainage; Favorable, mRS 0–3; Unfavorable, mRS 4–6 outcomes*.

### Comparisons of Long-Term Therapeutic Effects

#### Comparison of the Survival Percentage and the Survival Advantage Between the Two Groups

**Figure 5:**
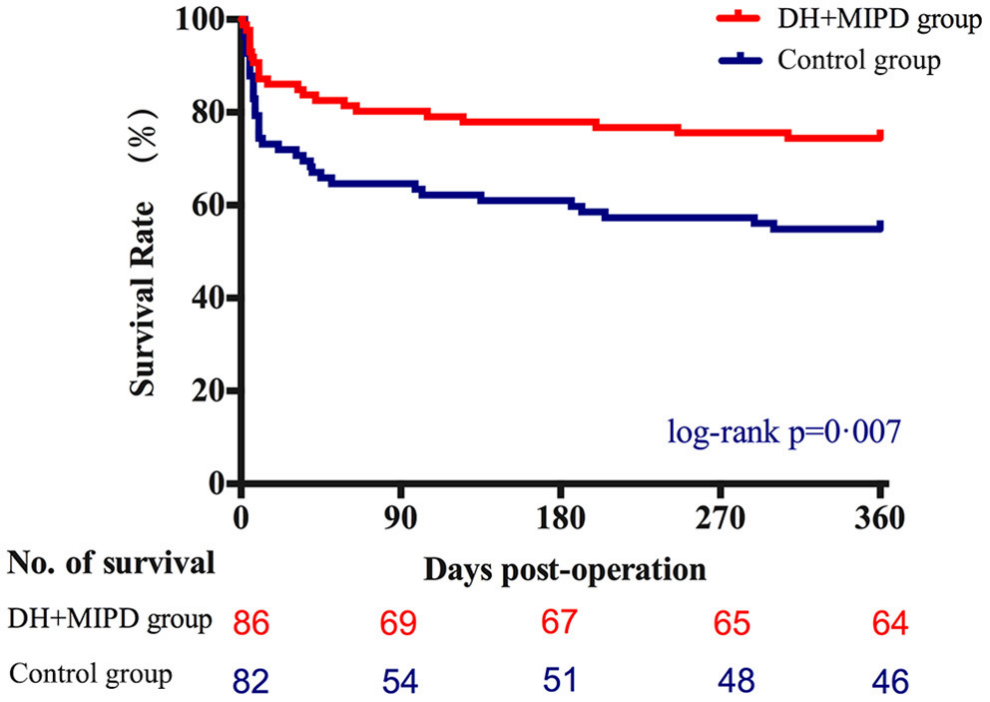
Comparison of survival percentage between the DH + MIPD group and the control group within 365 days post-operation. DH + MIPD, decompressive hemicraniectomy + minimally invasive puncture and drainage.

**Figure 6:**
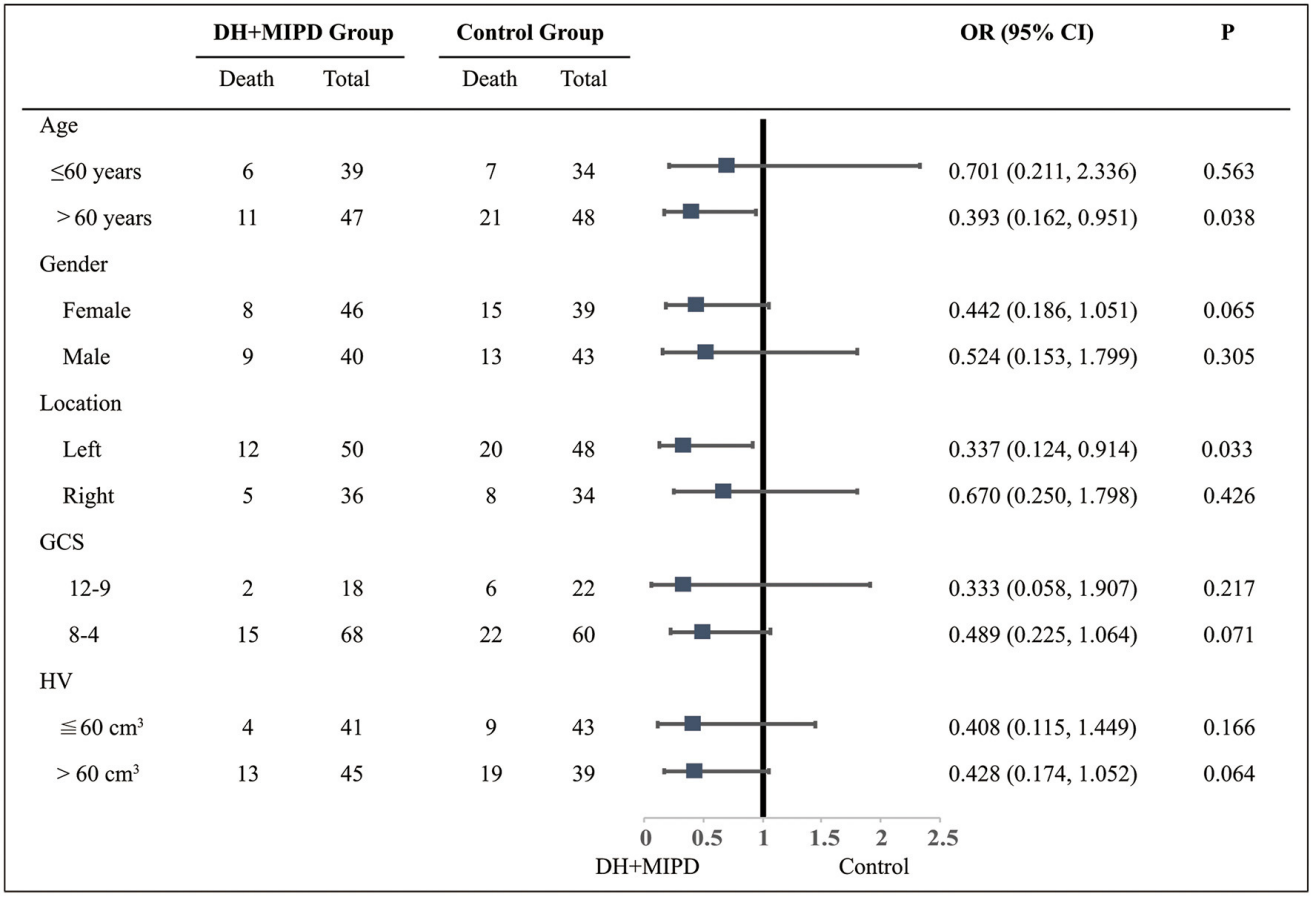
Subgroup analysis about the therapeutic effects of the two groups within 90 days post-operation. DH + MIPD, decompressive hemicraniectomy + minimally invasive puncture and drainage; GCS, Glasgow Coma Scale; HV, hematoma volume; OR, odds ratio.

Through the analysis of survival time, the results showed that the survival rate curve of the two groups both presented a sharp decline in the first 90 days and kept a gradual decline thereafter (Figure 5). Moreover, the survival percentage of the DH + MIPD group was more significantly higher than that of the control group (*P* = 0.007). Lastly, the 1-year survival rate of ICH patients in this study was 74.42% in the DH + MIPD group and 56.10% in the control group, respectively. Based on mortality within 90 days post-operation, a subgroup analysis showed that the patients in the two pre-specified subgroups had a definite survival advantage by using the DH + MIPD method; one subgroup was older, >60 years (*P* = 0.038), and the other one had hematoma located in the left dominant hemisphere (*P* = 0.033) (Figure 6).

#### Comparison of the Functional Outcome Between the Two Groups

**Figure 7:**
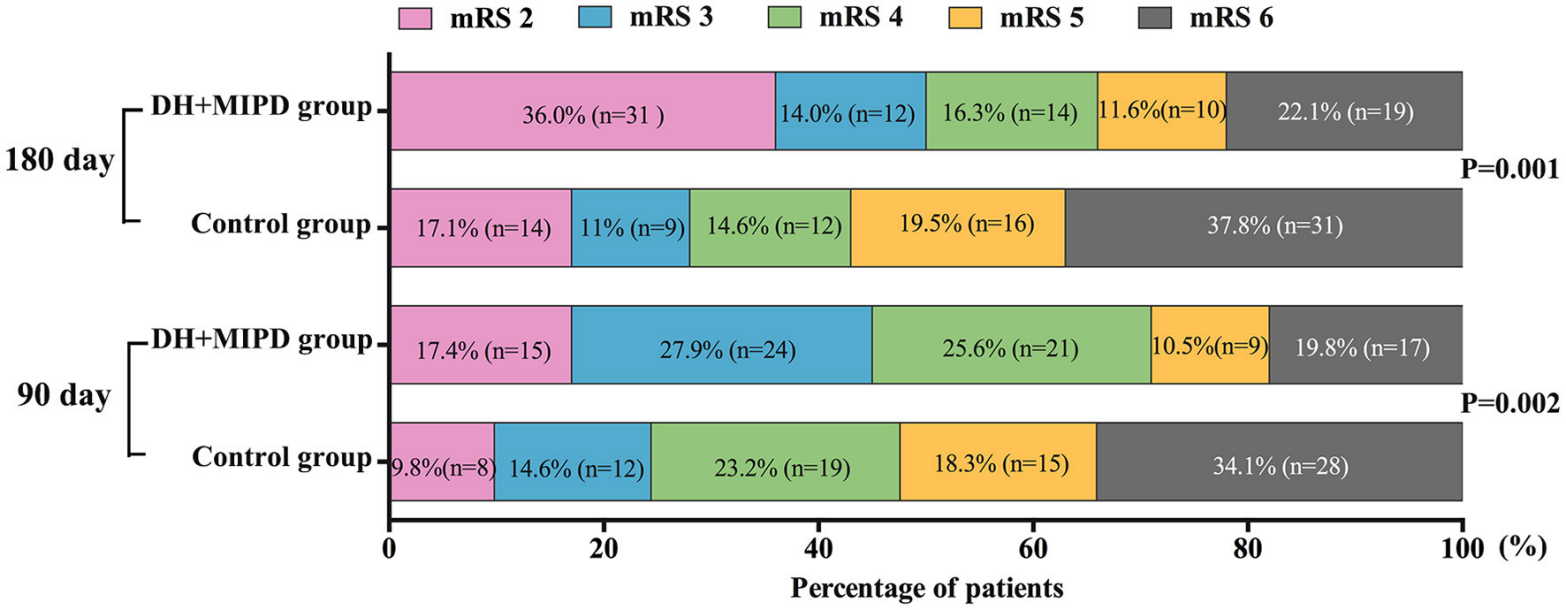
Comparison of mRS of the two groups within 90 and 180 days post-operation. DH + MIPD, decompressive hemicraniectomy + minimally invasive puncture and drainage; mRS, modified Rankin Scale.

Through the analysis of prognosis-based mRS, the distributions of mRS scores were significantly different in both groups at 90 days (*P* = 0.002) and at 180 days post-operation (*P* = 0.001) (Figure 7). The favorable outcome (mRS 0–3) rates in the DH + MIPD group were especially significantly higher than those in the control group at 90 days (45.3 vs. 24.4%, *P* = 0.004) and at 180 days post-operation (50.0 vs. 28.0%, *P* = 0.004) (Table 2).

## Discussion

Increased ICP is the core problem in the treatment of many neurological diseases. For the critical ICH patients with average HV of more than 60 ml, how to effectively reduce ICP to save patients' lives and ameliorate their functional prognosis was the key point of our experiment. To resolve this issue, our team took two effective measures to reduce ICP synergetically, combining DH with MIPD, assisted by urokinase to dissolve blood clots. Under the guarantee of satisfactory reduction of ICP, the whole experimental process presented its feasibility of hematoma evacuation and finally realized the hypothesis of reducing mortality and improving functional recovery.

The feasibility of hematoma evacuation by DH + MIPD method included two aspects: effectiveness and safety. The results (Figures 2–4) showed that the characteristic of hematoma evacuation was a gradual process in the DH + MIPD group, different from the quick removal in the control group. Under the action of urokinase, the ER of hematoma in the DH + MIPD group could finally reach up to 87% at 5 days post-operation. Importantly, during the course of hematoma removal, the change process of PHE in the DH + MIPD group showed such characteristics of less degree of edema, shorter duration of peak period, and more rapid edema regression. Under the cooperation of the decompressive effects of DH, the average ICP of the DH + MIPD group was significantly lower than that of the control group, which kept fluctuating below 15 mmHg all the time. In addition, the analysis of the operation-related clinical parameters, such as shorter duration of surgery, less blood loss, shorter length in the ICU, and lower mortality, all presented the advantages of DH + MIPD in effectively removing hematoma.

The security of DH + MIPD involves the following aspects; Firstly, DH and MIPD were both conventional and mature operative techniques, respectively, and the safety of MIPD assisted with thrombolysis therapy had been proven in many studies (8–14). Different from other studies that applied CT to guide the puncture, we adopt real-time ultrasound guidance in the whole process, which could help keep away from important blood vessels and ensure that the tip of the catheter was located in the desired position within the hematoma cavity. Secondly, during the whole process of the operation, the DH + MIPD method made a minimal injury to the cerebral tissue of the patient, only a minor puncture channel with a diameter of 2.6 mm on the cerebral cortex, and avoided frequent and excessive stretching and electrocoagulation of cerebral tissue in the control group, which might be a primary reason for the significant differences in PHE evolution post-operation between the two groups. Thirdly, continuous ICP monitoring for 5 days after the operation was more conducive to the judgment of intracranial conditions so as to make timely and judicious treatment for abrupt changes of the patients' condition (15). Fourthly, there were no differences in the incidence of complications between the two groups. The above-mentioned points jointly guaranteed the safety of DH + MIPD.

The assessment of the long-term results of the therapeutic effects involved the survival and the functional outcomes between the two groups. The survival rate of the DH + MIPD group was significantly higher than that of the control group within 1 year post-operation, with 74.42% in the DH + MIPD group and 56.10% in the control group, respectively (Figure 5). A subgroup analysis pointed out that the DH + MIPD method had a definite survival advantage for critical ICH patients older than 60 years old and with hematoma located in the left dominant hemisphere. The reasons for this phenomenon lay in the fact that the DH + MIPD method itself had irreplaceable features: shorter operation time, less blood loss, minimal injury to the cerebral tissue during operation (especially important for the dominant hemisphere), satisfactory hematoma removal, less degree of edema, better effect of decreasing ICP, and so on. Hegde et al. (16) also provided a similar view that surgical evacuation of spontaneous ICH had a definite survival advantage by delayed PHE.

Functional recovery is crucial to the postoperative quality of life in ICH patients. Our study demonstrated that the functional outcome of the DH + MIPD group was significantly better than that of the control group. With 6 months post-operation as the golden period of rehabilitation, the favorable outcome rate of the two groups had made a certain progress, owing to individualized and intensive functional exercise (17) associated with acupuncture and physiotherapy from traditional Chinese medicine. The favorable outcome rate of the DH + MIPD group increased from 45.3% at 90 days post-operation to 50.0% at 180 days post-operation. Meanwhile, that of the control group also increased from 24.4% at 90 days post-operation to 28.0% at 180 days post-operation. Certainly, a small portion of patients in mRS grade 4–5 died of complications due to long-term bed rest and improper care. Although there was a certain heterogeneity among various ICH studies, our result of reducing mortality and improving functional recovery was still consistent with those of most other studies on MIPD plus thrombolysis therapy (8–12, 18, 19). However, an open-label, multicenter, multinational, randomized clinical trial, the minimally invasive catheter evacuation followed by thrombolysis (MISTIE) III trial, made the conclusion that MISTIE was safe, but it did not improve the long-term functional outcome and cannot be recommended as routine care in patients suffering from supratentorial ICH (14). The differences between this conclusion and our result may be caused by the following four points: Firstly, the preoperative consciousness level of the patients in the two trials was different, with 26% of GCS 3–8, 44% of GCS 9–12, and 30% of GCS 13–15 in the MISTIE III trial compared with 79.1% of GCS 3–8 and 20.9% of GCS 9–12 in our trial. Secondly, the preoperative average HV was different, 45.8 ml in the MISTIE III trial vs. 65.6 cm^3^ in our trial. Thirdly, based on the previous two points, the surgical methods were different in the two trials (only a burr hole on the skull without DH in the MISTIE III trial). Lastly, the post-operative rehabilitation treatment was not clearly stated in the MISTIE III trial, compared with the individualized and intensive functional exercise in our trial. Therefore, it was reasonable that the differences in the patients' consciousness level, HV, operation method, and rehabilitation treatment resulted in different results.

In recent years, some trials (20–24) focused on DH with or without hematoma evacuation had been studied for ICH patients, and the results showed that DH without hematoma evacuation might reduce mortality and improve functional outcomes in certain populations of ICH patients. However, owing to the not randomized, controlled design and the smaller sample size in these trials, the results were controversial and needed further demonstration. Interestingly, Hayes et al. (25) made another research on hematoma evacuation with or without DH for ICH patients and held the view that hematoma evacuation with DH might improve the clinical outcome. Anyway, DH plays a very important role in decreasing ICP for larger spontaneous ICH in the acute phase.

Although this research has achieved some encouraging results at the present stage, many aspects still need to be further studied, owing to the limits of the retrospective trial. Firstly, our team will actively carry out multi-center research in order to better analyze the progress of hematoma, PHE, and ICP through larger sample sizes and expect that more severe ICH patients could benefit from the DH + MIPD method. Secondly, we will attempt to adjust the dosage and the frequency of urokinase, hoping to shorten the duration of hematoma drainage under the premise of ensuring safety. Thirdly, we will deeply explore the relationship between PHE and long-term outcomes while investigating whether targeted therapy focused on PHE, such as anti-inflammatory therapies (26, 27), could better promote the recovery of postoperative patients in the DH + MIPD group. Lastly, we will investigate the relationship between the repair of white matter fiber bundles and the functional recovery of patients at different time points.

## Conclusion

The role of early surgery for hematoma evacuation after ICH remains a topic of hot debate. There is no doubt that the operation remains the only life-saving measure in critical situations. Therefore, according to those serious ICH patients with refractory ICP, our study proved the feasibility of the DH + MIPD method on hematoma evacuation. Meanwhile, this new combination also implicated its significant advantages of minimal injury to cerebral tissue, satisfactory hematoma removal, less degree of edema, better effect of decreasing ICP, shorter operation time, less blood loss, and lower mortality (compared with the control group) and finally realized the possibility of reducing mortality and improving functional recovery. This method should be strongly recommended to critical ICH patients, providing one more choice for individualized therapy of ICH in the basal ganglia region.

## Data Availability Statement

The original contributions presented in the study are included in the article/supplementary materials, further inquiries can be directed to the corresponding author/s. The raw data supporting the conclusions of this article will be made available by the authors, without undue reservation.

## Ethics Statement

The studies involving human participants were reviewed and approved by Ethics Committee of the Second Affiliated Hospital of Chongqing Medical University. The patients/participants provided their written informed consent to participate in this study. Written informed consent was obtained from the individual(s) for the publication of any potentially identifiable images or data included in this article.

## Author Contributions

YC, JC, and GZ conceived, designed the study and provided suggestions for revising the manuscript. ZX, NH, and QY performed the experiments and provided suggestions for revising the manuscript. WC conducted the statistical analysis and provided suggestions for revising the manuscript. QH designed, drafted, revised, and gave final approval for the manuscript. All authors contributed to the article and approved the submitted version.

## Conflict of Interest

The authors declare that the research was conducted in the absence of any commercial or financial relationships that could be construed as a potential conflict of interest.
